# 

**Published:** 1935

**Authors:** 


### The Medical Library of the University of Bristol,

WITH WHICH IS INCORPORATED

**The Library of the Bristol Medico-Chirurgical Society.**

---

*The following donations have been received since the publication of the list in June,* 1935.

Sept. 1935.

| | |
|---|---|
| Mrs. Elizabeth F. Dall (1) .. .. .. | 1 volume. |
| Professor E. Fawcett (2) .. .. .. .. | ,, |
| Dr. John F. Fuller (3) .. .. .. .. | ,, |
| Dr. E. J. Hamlin (4) .. .. .. .. | ,, |
| Professor E. W. Hey Groves (5) .. .. | ,, |
| Isador Abrahamson Fellowship (6) .. .. | ,, |
| Liège University (7) .. .. .. .. | ,, |
| Professor C. Lloyd Morgan (8) .. .. .. | 6 volumes. |
| Dr. R. S. S. Statham (9) .. .. .. | 1 volume. |
| Sir Malcolm Watson (10) .. .. .. | 20 volumes. |

Unbound periodicals have also been received from Professor E. W. Hey Groves, Professor C. Bruce Perry, and Dr. J. Odery Symes.

### THE ONE HUNDRED AND FIFTY-SECOND LIST OF BOOKS.

The figures in round brackets refer to the figures after the names of the donors. The books to which no such figures are attached have either been bought from the Library Fund or received through the *Journal.*

**Abrahamson, I.** .. .. *Lethargic Encephalitis* .. .. .. .. (6) 1935

**Ballance, Sir C.** .. *The Conduct and Fate of the Peripheral Segment of a Divided Nerve in the Cervical Region, when united by Suture to the Segment of another Divided Nerve* .. .. 1934

**Bell, Sir C.** .. .. *The Hand* .. .. .. .. .. .. .. .. .. (8) 1834

**Berkeley, Sir C., and Bonney, V.** *Textbook of Gynæcological Surgery* 3rd Ed. 1935

**Bryce, A.** .. .. .. .. *Ideal Health* .. .. .. .. .. .. 3rd Ed. 1935

**Burnett, G. T.** .. .. *Outlines of Botany* .. .. .. .. .. .. (9) 1835

**Dall, W.** .. .. .. .. *Ground Porcelain Inlays* .. .. .. .. (1) 1933

**Fulton, J. F.** .. .. *A Bibliography of two Oxford Physiologists, Richard Lower . . . [and] John Mayow* (3) 1935

**Geddes, P., and Thomson, J. A.** *The Evolution of Sex* .. .. (8) 1899

*Gould's Medical Dictionary.* Edited by R. J. E. Scott . . . and C. V. Brownlow .. .. .. 4th revised Ed. 1935

**Ikin, A. G.** .. .. *Religion and Psychotherapy, a Plea for Co-operation* .. .. .. .. .. .. .. 1935

**Kerr, D. J. A.** .. .. *Forensic Medicine* ... .. .. .. .. .. .. 1935

**Logie, H. B.** (Ed.) *Standard Classified Nomenclature of Disease* 1935

**Morgan, O. G.** .. .. *Ophthalmology in General Practice* .. .. 1935

**Moureau, P.** .. .. *Contribution à l'Étude des facteurs d'individualisation du sang humain* (7) *Thèse* .. 1935

**Rolleston, G.** .. .. *Scientific Papers and Addresses.* Arranged and edited by W. Turner. 2 Vols. .. (8) 1884

**Ross, Sir R.** .. .. 20 volumes of his miscellaneous works (10)

**St. Mark's Hospital, London.** *Collected Papers . . ., Centenary Volume, 1835–1935* .. .. .. .. .. .. .. .. 1935

**Schrader, H.** .. .. *Economic Aspects of the Design of Sewage Treatment and Disposal Works* .. (4) 1934

**Siegel, M.** .. .. .. .. *Constructive Eugenics and Rational Marriage* [1934]

**Thomson, J. A.** .. *Outlines of Zoology* .. .. .. 4th Ed. (8) 1906

**Tuke, D. H.** .. .. *Sleepwalking and Hypnotism* .. .. .. (8) 1884
edited by W. Turner. 2 Vols. .. (8) 1884

### TRANSACTIONS, REPORTS, JOURNALS, ETC.

Collected Papers of the Mayo Clinic and Mayo Foundation. Vol. XXVI., 1934 .. .. .. .. .. .. .. .. .. .. .. .. .. .. 1935

Commission on Medical Education : Medical Education and Related Problems in Europe .. .. .. .. .. .. .. .. .. .. (2) 1930

Journal of Laryngology and Otology. Index Vols. XXXVI.—XLV. (1921–1930) .. .. .. .. .. .. .. .. .. .. .. .. .. 1932

Royal College of Surgeons, England : List of Transactions, Periodicals and Memoirs in the Library of the Royal College of Surgeons, England .. .. .. .. .. .. .. .. .. .. 2nd Ed. (5) 1931

Transactions of the Medical Society of London. Vol. 58 .. 1935

### Publications Received

From EDWARD ARNOLD & CO. :

*Medicine for Nurses.* By W. GORDON SEARS. Price 8s. 6d.

From J. W. ARROWSMITH LTD. :

*The "Albatross," being the Biography of Conrad Fitz-Gerald, M.R.C.S., L.S.A.* Written by C. T. FITZ-GERALD, Jun. Price 8s.

From JOHN BALE, SONS & DANIELSSON :

*Diabetes Mellitus : a Clinical Study.* By T. H. OLIVER, M.A., M.D., F.R.C.P. Price 3s. 6d.

*Manipulative Methods in the Treatment of Functional Disease.* By E. L. HOPEWELL ASH, M.D. Price 2s. 6d.

From CASSELL & CO. LTD. :

*Rheumatic Diseases : their Recognition and Treatment.* By FRANCIS BACH, M.A., M.D. Price 12s. 6d.

From HARPER & BROS. :

*Mental Health : its Principles and Practice.* By F. E. HOWARD, Ph.D., and F. L. PATRY, M.D. Price 15s.

From WILLIAM HEINEMANN LTD. :

*The Osteopathic Lesion.* By G. MACDONALD, M.B., Ch.B. (Edin.), D.O. (Kirksville, U.S.A.), and W. HARGRAVE-WILSON, D.O. (Kirksville, U.S.A.). Price 7s. 6d.

From HUTCHINSON & CO. :

*The Theory and Practice of Anæsthesia.* By M. D. NOSWORTHY, M.A., M.D., B.Ch., with a foreword by I. W. MAGILL. Price 12s. 6d.

From H. K. LEWIS & CO. LTD. :

*Reports on Chronic Rheumatic Diseases. . . .* No. 1. Ed. by C. W. BUCKLEY, M.D., F.R.C.P. Price 12s. 6d.

*Infections of the Urinary Tract.* By T. E. HAMMOND, F.R.C.S. Price 10s. 6d.

*An Anthology of Wit.* By ALEX E. ROCHE, M.A. Price 1s.

*A Guide to the Surgical Paper, with Questions and Answers.* By R. J. MCNEILL LOVE, M.S.(Lond.), F.R.C.S. (Eng.). Price 5s.

From PARKE, DAVIS & CO. :

*Vaccine and Serum Therapy.* (Compiled by members of the Staff of the Inoculation Department of St. Mary's Hospital, London, the introductory chapters having been written by Sir A. E. WRIGHT, M.D., founder of the Department.)

From UNIVERSITY OF GLASGOW :

*Experimental and Clinical Researches on Angina Pectoris and its Surgical Treatment.* By RENE LERICHE, F.R.C.S. (Eng.), F.A.S.A. (Hon.), etc. Price 1s. 6d.

From JOHN WRIGHT & SONS LTD. :

*The Medical Annual General Index and Review for the Ten Years* 1925—1934. Price 12s. 6d.

*The Medical Cookery Book.* Compiled by DOROTHY SEWART. Price 2s. 6d.

*The British Journal of Surgery.* Vol. XXIII, No. 89. Price 12s. 6d.

### Local Medical Notes

**The Long Fox Memorial Lecture.**—The Long Fox Memorial Lecture will be delivered by Dr. A. MacDonald Critchley, M.B., F.R.C.P., on Tuesday, October 22nd, at 5.15 p.m., in the Physiological Lecture Theatre. Title : " The Occurrence of Pain."

APPOINTMENTS.

Professor J. A. Nixon, C.M.G., M.D., F.R.C.P., to Industrial Health Research Board of Medical Research Council.

**University of Bristol.**

T. B. Davie, M.D., M.R.C.P., Professor of Pathology.

A. R. Parkes, M.B., Ch.B., Lecturer in Anatomy.

H. R. Noltie, M.A., B.Sc., Lecturer in Physiology.

Dorothy Woodman, M.D., M.Sc., B.S., Lecturer in Pathology.

R. G. Paul, F.R.C.S., Clinical Lecturer in Surgery.

H. J. Orr-Ewing, M.C., M.D., F.R.C.P., Clinical Lecturer in Medicine.

C. H. G. Price, M.B., Ch.B., Assistant Curator of the Pathological Museum.

EXAMINATION RESULTS.

**University of Bristol.**—Students of the University have recently passed the following examinations :—

M.D.—*Pass :* A. C. Fisher.

**University of London.**—M.B., B.S.—*Second Examination. Pass :* G. H. C. Hughes.

**Conjoint Board.**—M.R.C.S., L.R.C.P. *Pass : In Physics :* D. J. Jones. *In Biology :* J. L. Reynolds, J. F. Williams. *In Anatomy :* R. T. Moore, E. A. Griffiths. *In Physiology :*

M. G. B. Thompson. *In Pharmacology and Materia Medica:* P. W. J. Parkes, Rotha H. Dalton, D. Rosser. *In Pathology and Medicine:* N. P. Eskell.* *In Medicine:* D. R. Gray,* Irene G. Hamilton.* *In Medicine and Surgery:* J. G. Field.* *In Pathology:* F. C. Collingwood, Ursula W. Wood, W. H. Hayes. *In Midwifery:* A. W. Woolley.

L.D.S., R.C.S.—*Final Examination.* (*Part I.*) *Pass:* J. T. S. Hutchins. (*Part II.*) D. J. Dymond.*

**Society of Apothecaries.**—L.M.S.S.A. *Pass: In Medicine:* K. P. Pauli.* *In Midwifery:* S. Roberts.*

* Completing Qualification.

